# Is the modified household food security survey (HFSS) questionnaire a practical tool for screening food insecurity? Evidence from northwest of Iran

**DOI:** 10.1186/s12889-020-09014-8

**Published:** 2020-06-08

**Authors:** Helda Tutunchi, Mehrangiz Ebrahimi-Mameghani, Mohammad Asghari-Jafarabadi, Nazila Farrin, Sirous Tabrizi, Elnaz Vaghef-Mehrabany, Alireza Ostadrahimi

**Affiliations:** 1 Nutrition Research Center, Department of Clinical Nutrition, School of Nutrition & Food Sciences, Tabriz University of Medical Sciences, Tabriz, 5166614711 Iran; 2 Social Determinant of Health Research Center, School of Nutrition & Food Sciences, Tabriz University of Medical Sciences, Tabriz, Iran; 3 Road Traffic Injury Research Center, School of Health, Tabriz University of Medical Sciences, Tabriz, Iran; 4 Nutrition Research Center, School of Nutrition & Food Sciences, Tabriz University of Medical Sciences, Tabriz, Iran; 5 Faculty of Education, University of Windsor, Windsor, Canada

**Keywords:** Food insecurity, Short questionnaire, Food consumption, Socio-economic status, Social health, Iran

## Abstract

**Background:**

Adequate supplies of food and food security (FS) are the fundamental aspects of human societies, and considered one of the pivotal factors of individual and social health. The aim of the present study was to assess the applicability of the short questionnaire for screening food insecurity (FI) and to evaluate the prevalence of FI in northwest of Iran.

**Methods:**

In this study, 550 subjects aged ≥16 years were studied. Three-day dietary records and a short questionnaire were applied to estimate the prevalence of FI in terms of hunger and hidden hunger. The sensitivity, specificity and accuracy of the short questionnaire were assessed. Moreover, the association between the results of the short questionnaire and the criteria that were theoretically related to FI were examined. Data were presented as mean (SD), median (min-max) for the numeric normal and non-normal variables, respectively, and frequency (percent) for categorical variables. The between-group comparisons of variables were done using independent samples t test. P values less than 0.05 were considered statistically significant.

**Results:**

The prevalence of hunger and hidden hunger was 30.8 and 46.0%, respectively. Overall, 23.2% of the subjects were classified as “food secure”. The sensitivity, specificity and accuracy of the short questionnaire in determining hunger were 92.8% (95% CI: 87.3–95.9), 84.2% (95% CI: 79.3–89.3), and 87% (95% CI: 84–90.2), respectively. These values for hidden hunger were 21.6% (95% CI: 15.7–29.9), 92.3% (95% CI: 88.7–99.4), and 53.4% (95% CI: 47.9–59.8), respectively. Our study showed a statistically significant association between FI and socio-economic status. FI significantly enhanced the risk of underweight, while it markedly reduced the risk of overweight and obesity. The average frequency of monthly consumption of meat, dairy products, fruits, vegetables, and rice was significantly lower in food insecure group, while the median frequency of bread consumption was markedly higher in food insecure group. The participants of insecure group were less likely to consume fruits, vegetables, dairy products, rice and meat.

**Conclusions:**

FI was frequent in North-west of Iran. The findings indicated that the short questionnaire was a simple, low-cost and practical tool for screening FI in terms of hunger.

**Trial registration:**

IR.TBZMED.REC.1397.400.

## Background

Adequate supplies of food and food security (FS) are the fundamental aspects of human societies, and considered one of the pivotal factors of individual and social health [1, 2]. FS is the result of efficient food system operating. An efficient food system positively affects all aspects of FS [3]. The four dimensions of FS are food availability, access to food, utilization, and stability. Food availability indicates if sufficient food is readily at people’s disposal. Access to food makes sure whether all people have enough resources to supply the food they require. Utilization includes the food preparation, food distribution, water, sanitation, and health care practices. Stability is another dimension of FS, which guarantees continuation of the three other dimensions over time [4].

Food insecurity (FI) is defined as “*limited or uncertain access to nutritionally enough and safe foods, or limited ability to acquire acceptable foods through socially admitted ways*” [5, 6]. It is estimated that more than 852 million people worldwide do not have access to sufficient food for healthy and active lives; approximately nine million of these people are in developed nations and the remaining part live in the developing countries [7]. There is evidence indicating that about 20% of the Iranian population suffers from an inadequate dietary intake of energy and protein, while micronutrient deficiencies appears to be much higher [8, 9] . This condition can negatively affect quality of life, and adversely influence physical, social and mental well-being [10–12]. Therefore, providing useful and practical tools for assessment of FS is required to help with screening FS and consequently taking appropriate measures accordingly [8, 13]. Various indirect indicators, such as income, food consumption and nutritional situation have been previously used to monitor FI status in different countries [14–18]. Furthermore, well-designed questionnaires which are considered to be reliable, simple and low cost resources have been applied for direct assessment of FS [15, 19–28]. Radimer/Cornell questionnaire (12-item) has been found to be a highly reliable tool for assessing FI in Households with children [25]. In addition, one of the widely used measures of FS status is the 18-item Household Food Security Survey (HFSS) [20, 26]. Blumberg et al. [20] described an abbreviated version of the HFSS consisting of only six items (short questionnaire). They found that the short questionnaire may be adequate to identify a household’s level of FS when survey resources do not permit use of the full scale. They suggested that this measure maintains many of the essential indicators of FS, despite its abbreviated form [20]. For example, this questionnaire does not rely exclusively on specific measures of intake insufficiency. Other indicators capture self-perceived nutritional inadequacy, household food depletion, disrupted eating habits, and the repetitive pattern of decreased food consumption [20]. Collectively, Blumberg et al. [20] indicated that this form was a short but potentially valid and sufficiently beneficial instrument for national surveys and some state/local programs. Due to the high prevalence of FI in Iran, the use of simple, low-cost, rapid and useful tool for assessing FS status is necessary. Most of the previous studies have indicated that it is essential to determine the applicability of questionnaires in every country they are to be applied. This is due to the social and cultural diversities in various countries [8, 22, 29–31]. Accordingly, the aim of the present study was to assess the applicability of the short questionnaire (adapted from the HFSS questionnaire) for screening FI and to evaluate the prevalence of FI in northwest of Iran.

## Methods

### Study population and sampling

**Fig. 1:**
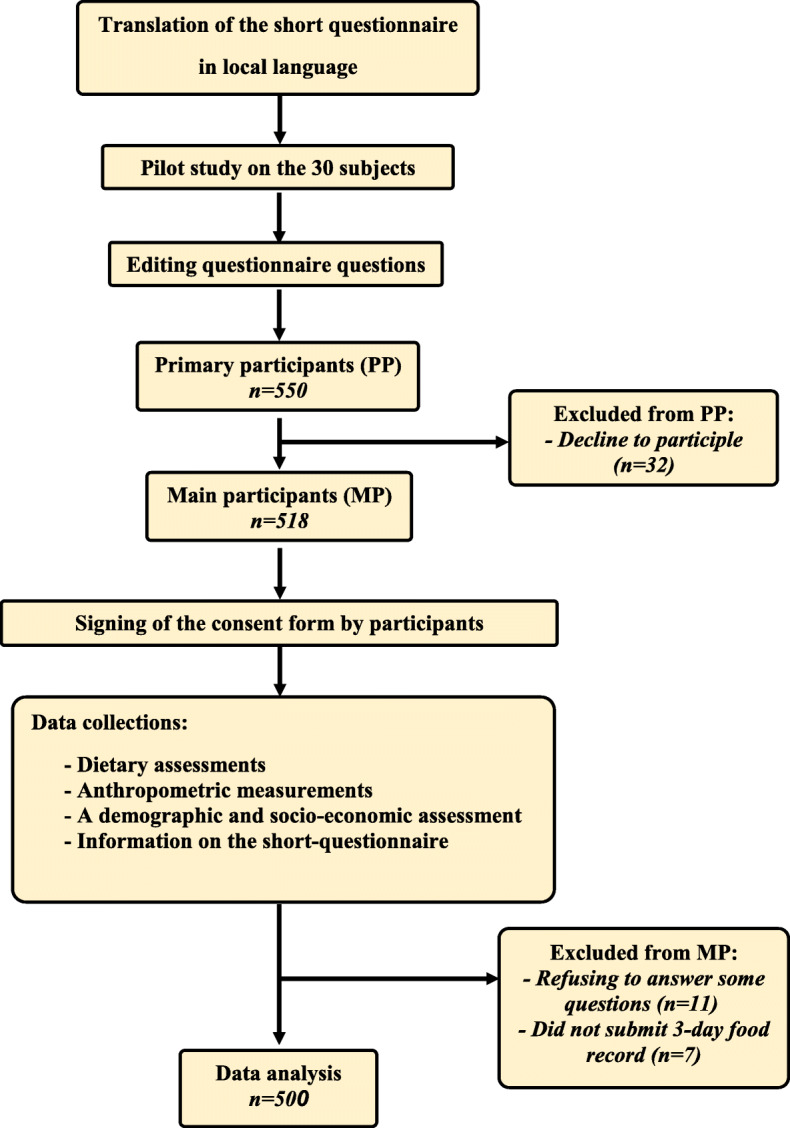
Flow chart of the study from the baseline until the end of study

In this cross-sectional study, the target area was a large industrial company in Tabriz which located in northwest of Iran. The study population consisted of 550 male and female workers, mainly the heads of the households (82.18%) selected by simple random sampling. According to the latest studies, the prevalence rate of FI is reported to be 49.2% in Iran [14]. By considering the prevalence of FI, confidence interval (CI) of 95% and power of 90%, the minimum sample size was calculated to be 384 subjects. However, a large sample size was considered to make the results more reliable. Although there was regular follow-ups and encouragement of individuals to participate in this study, the rate of participation was 90%. Five hundred subjects, including 285 males and 215 females (16+ years) completed the study (Fig. 1).

Eligible participants signed a consent form which described the study protocol in detail. This study was approved by the Nutrition Research Center of Tabriz University of Medical Sciences, Tabriz, Iran. Data were collected on age, gender, family size (the number of people who lived together in the same house), body mass index (BMI), the educational level of the head of the household, the occupation of the head, the annual per capita income, and dietary assessment via a face-to-face interview with the participants of the study.

### Anthropometric measurements

Body weights and heights were measured using a Seca scale (Seca, Germany) and a stadiometer (Seca, Germany), respectively. The individuals were measured barefoot, wearing light clothing. BMI was calculated by dividing weight by squared height (kg/m^2^).

### Determination of the educational and occupational positions of the household head

Information related to the educational and occupational positions of the head of households was collected through a questionnaire. In order to determine the occupational position of the household head, jobs were categorized into four career levels: high-level (i.e., high-ranking specialist, executive manager, senior employee, and high-paying jobs), medium-level (i.e., non-degree specialist, foreman, and serviceman jobs), low-level (i.e., workers, low-skilled jobs, and certain low-income jobs), and lowest-level (i.e., agricultural workers, retail sales clerk, maintenance services, and other low-income jobs). The occupational and educational positions of the heads of the households who were not employee of this factory were also collected using this questionnaire through interview with a member of their households who participated in this study.

### Information related to annual per capita income

Household income data was obtained by a questionnaire. The purpose of this questionnaire was to calculate the net income of the households in the research year, including wage and salary earnings from public, private, or cooperative sectors, and miscellaneous income (any income received outside of typical employee wages). The annual per capita income was calculated through dividing the total net income of the household members by the number of family members.

### Dietary assessment

Information on food consumption was obtained by a three-day food record (two weekdays and one weekend; non-consecutive) and compared to the Dietary Reference Intakes (DRIs). The dietary records were based on estimated values in household measurement. Subjects were instructed by a trained dietitian to consume their usual diet. Moreover, they were trained on how to fill out the food diary. Upon the form completion, an in-person interview was conducted with the study subjects to make sure that the foods recorded were typical of their routine diet. Then, data on food intakes were analyzed by Nutritionist IV software (First Databank, San Bruno, CA, USA) modified for Iranian foods. The dietary variables included intakes of energy and selected four key nutrients (protein, vitamin A, vitamin B_2_, and calcium).

The study Participants also completed a validated food frequency questionnaire (FFQ) [32]. The FFQ, a Willett-format questionnaire modified according to Iranian food items, contained questions about frequency of food intake for 168 food items during the past year. The food items were chosen according to the most frequently consumed items in the national food consumption survey in Iran. The FFQ included six groups: cereals, grains, meat, dairy products, vegetables and fruits.

### Information on the short-questionnaire

The short questionnaire of the HHFS consisted of six items [20]. In the first step, permission was obtained from the authors of this questionnaire for using their scale and then the questions of this form were translated into local language. A pilot study was conducted first, to validate the questions of this short form. To do so, the questionnaire was pre-tested on 30 subjects who were similar in major characteristics to the main study sample. Then the questions were edited and the final version of the questionnaire was used to assess FS of the individuals in the main part of the research. Based on the short questionnaire, responses of ‘often’ or ‘sometimes’ on questions Q5 and Q6 and ‘yes’ on Q1, Q3 and Q4 were coded as affirmative (yes). Responses of ‘almost every month’ and ‘some months but not every month’ on Q2 were coded as affirmative (yes). Participants who gave positive answers to two or more questions were considered as food insecure [20].

### Assessing the food insecurity based on dietary records

In general, key nutrients are mainly used in evaluating optimal and adequate food intake patterns. According to the available data, key nutrients include protein, calcium, vitamin A, and vitamin B2 in Iran [33, 34]. To determine the prevalence of FI based on data obtained from dietary records, two models were developed in terms of hunger and hidden hunger [33]. According to the first model, inadequate intake of energy was the indicator for assessing the prevalence of hunger. Based on this model, subjects whose energy intake was less than 90% of DRI, were classified as food insecure. In the second model, the indicators for assessment of the prevalence of hidden hunger were adequate consumption of energy and inadequate intake of one (or more) of the four key nutrients including protein, calcium, vitamin A, and vitamin B_2_. According to this model, people whose energy intake was more than 90% of DRI, but the consumption of one (or more) of four key nutrients was less than 80% of DRI, were classified as food insecure [33, 34].

### Assessment of the applicability of the short questionnaire

To assess the applicability of the short questionnaire, the correlation between the results of this form and the criteria theoretically related to FI was assessed. The criteria included the factors affecting FI (including income, the occupational position of the household head, the educational level of the head and the family size), as well as the consequences of household FI (BMI, and inappropriate frequency consumption of food groups). Multiple studies on dietary assessment indicated that food insecure subjects have energy shortage and micronutrient deficiencies [34–37]. Consequently, the data collected from the short questionnaire was compared with information provided by three-day dietary records which is considered as the gold standard for dietary intake assessment. According to the previous studies, socio-economic and demographic factors such as income, the occupational position of the household head, the educational level of the head and the family size are the most important determinants of household FI [38–41]. In addition, abnormal BMI and inappropriate frequency of food consumption are the consequences of FI [42–45]. Therefore, the effects of socio-economic and demographic factors on FI (as assessed by the short questionnaire) were also assessed. To investigate the effect of FI on BMI, FI distribution was assessed through BMI categories.

To assess the impact of FI on frequency of food groups intake, the food items used in the FFQ were summarized in order to identify and determine the food groups /items that were considered as a comparison criterion in the two food secure and insecure groups. The correlation between frequency of food consumption and monthly per capita income was measured using the Pearson correlation coefficient test. Because economic status is the most important factor determining FI [46], food groups that had a significant correlation with income, were chosen as comparison criteria in food secure and insecure groups. If there was no association between frequency of a food group consumption and income, the food item/items in that group was/were selected as the comparative criterion.

### Statistical analysis

The analyses were done using STATA software version 15 (StataCorp, College Station, Texas USA). Normality of the numeric variables was checked using Kolmogorov- Smirnov test. Data were presented as mean (SD), median (min-max) for the numeric normal and non-normal variables, respectively, and frequency (percent) for categorical variables. The between-group comparisons of variables were done using independent samples t test.

To assess the diagnostic accuracy of the short questionnaire based on hunger and hidden hunger status, diagnostic measures of sensitivity, specificity, accuracy, positive predictive value (PV+), negative predictive value (PV-), positive likelihood ratio (LR+), negative likelihood ratio (LR-) and the Cohen’s kappa coefficient along with their 95% CI as well as the odd ratio (OR) were computed by epitab procedure in STATA software. In all analyses, P values less than 0.05 were considered as statistically significant.

## Results

Table 1 shows the basic characteristics of the study participants. The study sample included 285 males (57%) and 215 females (43%), with mean age of 35.3 (SD 13.7) years. Total prevalence of overweight and obesity was 52.4% (95% CI: 46.1–58.3). In addition, 27.8% (95% CI: 21.9–33.4) of the participants were underweight. The average number of persons per household was 5.6 (SD 1.2) and the mean income per capita was $ 395 (SD 80.6). 19.6% (95% CI: 14.3–25.9) of the household heads had attended school for less than 5 years, and 13.0% (95% CI: 10.1–19.9) of them had the lowest level of occupations. Based on dietary records, the prevalence of hunger and hidden hunger in the area was 30.8% (95% CI: 25.8–39.2) and 46.0% (95% CI: 41.3–50.8), respectively. Overall, 23.2% (95% CI: 18.1–28.2) of the subjects were classified as food secures.

**Table 1:** Basic characteristics of the study participants

Characteristics	Number	Percent
**Gender**		
Females	215	43.0
Males	285	57.0
**Age**		
16–30	215	43.0
31–50	197	39.4
> 50	88	17.6
**Household size (number)**		
2–3	79	15.8
4–5	152	30.2
6–7	183	36.6
> 7	86	17.2
**Body mass index (BMI)**		
< 18.5	139	27.8
18.5–24.9	99	19.8
25–29.9	159	31.8
≥ 30	103	20.6
**Monthly income per capita ($US)**		
≤ 285	141	28.2
286–609	196	39.2
610–833	101	20.2
≥ 833	62	12.4
**Educational level of the household head (years)**		
< 5	98	19.6
6–11	273	54.6
> 12	129	25.8
**Occupation of the household head**		
Lowest level	65	13.0
Low level	179	35.8
Moderate level	213	42.6
High level	43	8.6

According to Table 2, in subjects who were insecure in terms of hunger, the average energy intake was 69.2% of DRIs (95% CI: 63.6–84.9). In subjects who were secure in terms of hidden hunger, the median intake of energy was 123.7% of DRIs (95% CI: 112.5–133.9) which was above the recommended amount (90–110% of DRIs) [9]. Furthermore, the average intake of dietary calcium in food insecure group was 68.7% of the DRIs (95% CI: 52.7–77.4), which was less than the recommended amount.

**Table 2:** Average of energy and key nutrition intakes compared to DRIS according to hunger and hidden hunger status

Food insecurity spectrum	Status	Energy (%95 CI)	Protein (%95 CI)	Vitamin A (%95 CI)	Vitamin B2 (%95 CI)	Calcium (%95 CI)
Hunger	Food secure	110.2 (98.5–113.7)	109.4 (94.5–119.4)	113.8 (90.1–133.6)	89.3 (77.5–101.7)	77.5 (59.5–98.6)
	Food insecure	69.2 (63.6–84.9)	78.9 (61.8–87.9)	91.4 (77.5–102.8)	68.6 (54.9–83.6)	49.9 (30.7–78.8)
Hidden hunger	Food secure	123.7 (112.5–133.9)	132.8 (127.5–139.7)	121.3 (114.1–144.5)	107.8 (101–127.3)	96.3 (90.5–102.9)
	Food insecure	105.5 (92.9–115.5)	112.3 (96.1–120.7)	108.9 (102.3–113.9)	84.2 (72.5–88.9)	68.7 (52.7–77.4)

*DRIs* dietary reference intakes

Affirmative responses to questions of the short questionnaire are shown in Supplemental Table 1. The findings demonstrated that more participants of the study (67.8%) gave affirmative answer to question six indicating that the majority of subjects could not afford to eat balanced meals. In addition, 39.4% of the participants gave the affirmative response to two or more questions and were considered as food insecure.

According to Table 3, Sensitivity, specificity and accuracy of short questionnaire for assessment of hunger were 92.8% (95% CI: 87.3–95.9), 84.2% (95% CI: 79.3–89.3), and 87% (95% CI: 84–90.2), respectively. These values were 21.6% (95% CI: 15.7–29.9), 92.3% (95% CI: 88.7–99.4), and 53.4% (95% CI: 47.9–59.8) for hidden hunger. Furthermore, the kappa agreement between two questionnaires in terms of hunger and hidden hunger was 76% (95% CI: 72–81, *p* <  0.0005) and 16% (95% CI: 11–29, *p* <  0.001), respectively. FI prevalence showed a positive association with household size (P for trend < 0.001), while there was a negative correlation between FI and income, educational level of the household head, and occupational position of the head (P for trend < 0.001; Table 4). FI significantly enhanced the risk of underweight (RR = 12.8, 95% CI: 7.8–23.9, *p* <  0.001), while it reduced the risk of overweight and obesity (RR = 0.39, 95% CI: 0.19–0.74, *p* <  0.001 and RR = 0.22, 95% CI: 0.11–0.68, *p* <  0.005, Table 5).

**Table 3:** Indicators of the short questionnaire based on hunger and hidden hunger status

Indicator	Hunger (95% CI)	Hidden Hunger (95% CI)
Sensitivity (%)	92.8 (87.3–95.9)	21.6 (15.7–29.9)
Specificity (%)	84.2 (79.3–89.3)	92.3 (88.7–99.4)
Accuracy (%)	87 (84–90.2)	53.4 (47.9–59.8)
False positive error rate (%)	15.6 (11.4–20.7)	7.6 (2.9–12.3)
False negative error rate (%)	7.1 (1.2–8.3)	78.8 (71.5–84.9)
Positive predictive value (pv+) (%)	72.5 (65.3–80.5)	76.9 (63.2–83.3)
Negative predictive value (pv-) (%)	96.3 (93.2–97.9)	49.3 (41.9–56.8)
Likelihood ratio positive (LR+)	5.94 (4.29–9.7)	2.84 (1–14.5)
Likelihood ratio negative (LR-)	0.08 (0.01–1.3)	0.85 (0.79–0.91)
Cohen’s kappa coefficient (κ)	0.76 (0.73–0.84)*	0.16 (0.11–0.33)**

*CI* confidence interval

*p <  0.0005, ** *p* <  0.001

**Table 4:** Odds ratios for food insecurity by demographic and socioeconomic status based on the short questionnaire

	n	%	OR** 95% (CI)
**monthly income per capita ($US)**			< 0.001 *
< 285	119	84.39	1.00 (reference)
286–609	63	32.14	0.51 (0.43–0.66)
610–833	11	10.89	0.18 (0.11–0.29)
> 833	4	6.4	0.12 (0.10–0.22)
**Educational level of the household head (years)**			< 0.001*
< 5	88	89.79	1.00 (reference)
6–11	84	30.76	0.34 (0.21–0.42)
> 12	25	19.37	0.25 (0.19–0.36)
**Household Size (number)**			< 0.001*
2–3	9	11.39	1.00 (reference)
4–5	40	26.31	2.47 (2.37–2.63)
6–7	84	45.90	4.21 (3.96–4.42)
> 7	64	74.41	6.78 (6.29–6.93)
**Occupational position of the household head**			< 0.001*
Lowest level	57	87.69	1.00 (reference)
Low level	85	47.48	0.61 (0.51–0.69)
Moderate level	51	23.94	0.34 (0.27–0.55)
High level	4	9.30	0.21 (0.12–0.39)

*n* number of individuals with food insecurity, *OR* odds ratio, *CI* confidence interval

**adjusted for age and gender

* P for trend

**Table 5:** Distribution of food insecurity status through BMI categories based on the short questionnaire

Status	BMI category			
	< 18.5	18.5–24.9	25–29.9	≥ 30
Food secure-no (%)	15 (4.95)	70 (23.10)	127 (41.91)	91 (30.03)
Food insecure-no (%)	124 (62.9)	29 (14.7)	32 (16.3)	12 (6.09)
Total-no (%)	139 (27.8)	99 (19.8)	159 (31.8)	103 (20.6)
RR^a^ (95% CI)	12.8 (7.8–23.9)**	0.64 (0.47–1.2)	0.39 (0.19–0.74)***	0.22 (0.11–0.68)***

*BMI* body mass index, *RR* relative risk, *CI* confidence interval

^a^adjusted for age and gender

**p <  0.001, *** *p* <  0.005

Association between the monthly income per capita and frequency of food groups / items is presented in Supplemental Table 2. The results indicated that consumption frequency of meat, dairy products, vegetable and fruit groups were directly correlated with monthly income per capita (*r* = 0.522, 0.309, 0.311, and 0.568 respectively, *p*-value < 0.01). Although the association between the consumption frequency of cereal and grain groups, and monthly income was insignificant (*r* = − 0.017 and − 0.073 respectively, *p*-value> 0.05), frequency of rice consumption was directly associated with income (*r* = 0.231, *p*-value < 0.05) and frequency of bread consumption was inversely correlated with income (*r* = − 0.421, *p* <  0.05).

The mean consumption frequency of meat, dairy products, fruits, vegetables, and rice was significantly lower in food insecure group than food secure group (*p*-value < 0.001), while the average frequency of bread consumption was higher in food insecure group compared to food secure group (*p*-value< 0.001) (Table 6). FI was significantly associated with a less frequent consumption of fruits, vegetables, dairy products, rice and meat (Supplemental Table 3).

**Table 6:** Mean values for weekly consumption frequency of food groups / items based on the food security status

Food groups/ items	Food secure (standard deviation)	Food insecure (standard deviation)	***P***-Value
Bread	14.93 (5.25)	18.39 (3.78)	< 0.001
Rice	6.93 (4.7)	3.2 (1.5)	< 0.001
Meat	6.5 (5.3)	1.9 (1.2)	< 0.001
Dairy Products	17.35 (8.89)	10.91(7.33)	< 0.001
Vegetables	23.65 (12.13)	16.22 (9.3)	< 0.001
Fruits	21.22 (14.23)	10.38 (9.68)	< 0.001

Values were adjusted for age and sex

## Discussion

This cross-sectional investigation was designed to evaluate the applicability of a short questionnaire for screening FI in northwest of Iran. In our previous study, we assessed some epidemiologic indicators of this short form questionnaire on the study population (*n* = 300) which was a different location in Tabriz (Asadabadi Medical Centre) [34]. While, in the present work, the target area was a large industrial company in northwest of Iran, with different people and larger sample size (i.e. *n* = 550). Moreover, in the current study, the correlation between the results of this questionnaire and the criteria theoretically related to FI was also assessed to determine the applicability of the short questionnaire in study population. In the study by Dastgiri et al. [34], dietary data was obtained using a 24-h food-recall questionnaire. Whereas we compared the data collected from the short questionnaire with information provided by dietary records in the present research.

The results of nutritional status based on dietary records in the study population revealed that the prevalence of hunger and hidden hunger was 30.8 and 46.0%, respectively. Overall 23.2% of the subjects were classified as food secure, indicating that FI was prevalent in the studied population. The meta-analysis of 31 studies in Iran demonstrated that the prevalence of FI was 49.2% in households, 67.0% in children, 61.0% in mothers, 49.0% in adolescents, and 65.0% in the elderly [14]. The previous studies on Iranian households have also shown that more than 20% of the population has insufficient energy and protein intakes, while micronutrient deficiency is estimated to be much higher [8, 47]. In addition, studies in Iran have demonstrated that the average intakes of vitamin B_2_, vitamin A, and calcium are lower than recommended [47, 48]. In the present study, the average consumption of dietary calcium in the food insecure group was 68.7% (95% CI: 52.7–77.4), which was less than the recommended value. In addition, in subjects who were secure in terms of hunger and hidden hunger, the average rate of energy intake compared to DRIs was 123.7%, which was higher than the recommended intake (90–110% of DRI). This finding might demonstrate that a number of food secure subjects have been able to meet their micronutrients’ requirements through overeating; in other words, adequate intake of micronutrients was the result of overeating rather than following a balanced diet. This might indicate inappropriate diet and eating habits in the study population. The prevalence of FI was 39.4% based on the short questionnaire, which was higher than that reported in the study by Dastgiri and colleagues in Iran [34]. In addition, in the study by Gulliford and his colleagues [19] who also applied this questionnaire, FI incidence was 25.2% in Trinidad and Tobago. In the current study, the percentages of affirmative answers to the six questions were 25, 16, 24, 18.6, 16.6, and 68.8%, respectively, which were higher than the those reported in the studies performed in the United States and Caribbean studies [19, 29]. The majority of the participants (68.7%) gave affirmative responses to question six, which was well above the values reported by other similar studies [29, 34]. The results of our study demonstrated that, although sensitivity, specificity, and accuracy of the short questionnaire were significantly low for surveillance of FI in terms of hidden hunger, the questionnaire can adequately efficiently detect hunger or severe FI. Furthermore, the degree of agreement between the results of the short questionnaire and dietary records was good for screening FI in terms of hunger. Conversely, the agreement was poor for assessing FI in terms of hidden hunger. In line with the present study, Dastgiri et al. [34] reported that the epidemiologic indicators of the short questionnaire were acceptably high for assessment of FI in terms of hunger in the area of their study. Low sensitivity and accuracy of the short questionnaire for screening FI in terms of hidden hunger might be attributed to the limited questions of the questionnaire. Moreover, economic status is one of the most significant determinants of FI, and the questions of this questionnaire have been designed in a way to focus on the financial constraints; as a result, other important factors remain overlooked in this regard. Note that significant groups of people who are food insecure in terms of hidden hunger, do have economic and physical access to foods, but have poor food choices most likely due to low nutrition literacy and lack of awareness in selecting a healthy and balanced diet. Consequently, the absence of questions targeting the subjects’ knowledge on healthy eating habits and balanced eating patterns in this questionnaire probably reduced the value of epidemiological indicators of the short form for assessing FI in terms of hidden hunger.

To examine the applicability of the short questionnaire, the association between the results of this form, and the criteria theoretically related to FI as well as the consequences of household FI were assessed. Consistent with previous findings, our study revealed an association between FI and demographic and socio-economic status in the study population. Similar to some previous results [17, 43, 49], FI prevalence showed a significant positive correlation with household size, while there was a statistically significant negative association between FI and income, educational level, as well as the occupational position of the household head. In relation to the association between FI and BMI, several studies have found that FI is linked to BMI [19, 34, 50–52]. Gulliford et al. [19] reported that FI was prevalent across all BMI levels. On the other hand, FI was significantly associated with underweight, but not with obesity. The findings of Dastgiri et al. [34] suggested that FI enhanced the risk of underweight and reduced the rate of overweight or obesity. In the study of Cheung et al. [53], FI was associated with increased BMI. A study demonstrated that although mild or moderate FI was associated with a higher rate of obesity, severe FI was related to the lower risk of obesity [54]. In the current study, FI significantly increased the risk of underweight while reducing the risk of overweight and obesity. Several studies have also shown that there is a correlation between FI and food consumption frequency [8, 46, 55]. In a study by Gulliford et al. [19], food insecure subjects were less likely to be frequent consumers of fruit or green vegetables or salads. In a recent study, FI negatively affected the fruit and vegetable consumption among families with individuals under 18 years [55]. Zerafati Shoae et al. [8] reported that as the severity of FI increased, the frequency of consumption of fruits, vegetables, dairy products, red meat and rice significantly diminished, while the frequency of consumption of potato and bread increased markedly. These observations are consistent with our results indicating that the mean consumption frequency of meat, dairy products, fruits, vegetables, and rice was significantly lower in the food insecure group, while the average consumption frequency of bread was significantly higher in the food insecure group compared to the food secure group. In addition, the subjects who were food insecure or unable to afford a balanced diet were less likely to eat fruits, vegetables, dairy products, rice, and meat.

Consistent with previous studies, the present research lends general supports to the applicability of the short-form questionnaire in screening FI in terms of hunger in Iranian social, economic, and cultural context. However, our findings indicated that the scale works poorly in assessing FI in terms of hidden hunger, and further research is required to improve the applicability of the scale in this regard. Additionally, our findings demonstrated that there was a strong correlation between the results of the short-form questionnaire and the criteria theoretically related to FI. Furthermore, in agreement with the results of the previous studies, the present research, using the short form, found that abnormal BMI could be one of the consequences of FI. On the other hand, inappropriate frequency of food consumption was also observed as a result of FI in the present study. On the whole, the findings of this study indicated that the short-form questionnaire was a simple, low-cost, rapid, and practical tool for screening severe FI. According to the high prevalence of FI, macroeconomic policies should be implemented and necessary measures must be taken to help people meet their nutritional requirements; these measures could include promoting the nutritional knowledge of household members as well as supporting the households [56].

Errors in food reporting and quantification can vary with the type of dietary methodology. The dietary record approach is often regarded as the “*gold standard”* in comparison to other self-report dietary assessment methods such as the 24-h dietary recall method. A major strength of our study was that three-day food records were used to determine dietary intakes of the participants. This study had some limitations too. Due to the cross-sectional design of the study, the generalizability of the samples to the population is limited. Moreover, the participants were not willing to announce their deprivation; especially lowest level individuals were rarely willing to express their food basket as too poor. Furthermore, we did try to interview with the head of households and we achieved it in most of the studied households. However, the person who were responsible for food preparation and replied to the questions were few and it was inevitable. Therefore, it could be considered as another limitation. As the final limitation of the study design, it was cross-sectional rather than prospective. Therefore, prospective studies are required to confirm these findings.

## Conclusions

It can be concluded that FI was frequent in north-west of Iran. The findings indicated that the short questionnaire was a practical tool for screening FI in terms of hunger. Although this questionnaire was a weak tool for assessing all dimensions of FI, it may be used as a simple, low-cost, rapid and practical tool for the screening and surveillance of severe FI in similar areas. Further investigations are needed to validate the instrument for national surveys and state/local programs.

## Supplementary information

**Additional file 1: Supplemental Table 1.** Affirmative responses to questions of the short questionnaire. **Supplemental Table 2.** Association between the monthly income per capita and frequency of food groups / items consumption. **Supplemental Table 3.** Consumption frequency of food groups/ items related to whether subjects could afford to eat balanced meals.

## Data Availability

The datasets used and/or analyzed during the current study are available from the corresponding author on a reasonable request.

## Abbreviations

BMI: Body Mass Index
DRIs: Dietary Reference Intakes
FFQ: Food Frequency Questionnaire
FI: Food Insecurity
FS: Food Security
HFSS: Household Food Security Scale
LR: Likelihood Ratios
OR: Odds Ratio
PV: Predictive Values
