# Supplementary material for: Is the modified household food security survey (HFSS) questionnaire a practical tool for screening food insecurity? Evidence from northwest of Iran
Source: BMC Public Health. 2020 Jun 8;20:883. doi: 10.1186/s12889-020-09014-8 (PMC7282109; doi:10.1186/s12889-020-09014-8)
Supplement: Supplementary file 1 — Additional file 1: Supplemental Table 1. Affirmative responses to questions of the short questionnaire. Supplemental Table 2. Association between the monthly income per capita and frequency of food groups / items consumption. Supplemental Table 3. Consumption frequency of food groups/ items related to whether subjects could afford to eat balanced meals. [file 12889_2020_9014_MOESM1_ESM.docx]

**Supplemental Tables**

**Supplemental Table 1**

**Affirmative responses to questions of the short questionnaire**

| **a** | **Affirmative responses to questions of the short questionnaire** | n (%) |
| --- | --- | --- |
| 1 | Did you ever cut the size of meals or skip meals because of lack of money for food in the last 12 months? (Yes, No) | 125 (25) |
| 2 | If yes, how often? (Almost every month, some months but not every month, only 1 or 2 months) | 80 (16) |
| 3 | Did you ever eat less than you felt you should because there was not enough money to buy food in the last 12 months? (Yes, No) | 120 (24) |
| 4 | Were you ever hungry but did not eat because you could not afford enough food in the last 12 months? (Yes, No) | 93 (18.6) |
| 5 | Food did not last, and did not have money to get more. (Was that often, sometimes, or never true for you in the last 12 months?) | 83 (16.6) |
| 6 | Could not afford to eat balanced meals. (Was that often, sometimes, or never true for you in the last 12 months?) | 339 (67.8) |
| **b** | **The sum of affirmative responses** |  |
|  | 0 | 128 (25.6) |
|  | 1 | 175 (35) |
|  | 2 | 56 (11.2) |
|  | 3 | 54 (10.8) |
|  | 4 | 36 (7.2) |
|  | 5 | 28 (5.6) |
|  | 6 | 23 (4.6) |

Yes” is the affirmative answer to questions 1, 3, and 4; “almost every month” and “some months but not every month” are affirmative responses to question 2; “often” and “sometimes” are affirmative responses to questions 5 and 6.

**Supplemental Table 2**

**Association between the monthly income per capita and frequency of food groups / items consumption**

| **Food groups/items** | **Pearson correlation coefficient** | **P-Value** |
| --- | --- | --- |
| Cereals Group | -0.023 | 0.34 |
| Bread | **-0.421** | **< 0.05** |
| Rice | **0.231** | **< 0.05** |
| Spaghetti | -0.036 | 0.22 |
| Grains Group | -0.073 | 0.61 |
| Lentils | -0.027 | 0.44 |
| Split peas | -0.039 | 0.79 |
| Peas | -0.063 | 0.10 |
| Beans | -0.029 | 0.79 |
| Miscellaneous | -0.134 | 0.55 |
| Meat Group | **0.522** | **< 0.001** |
| Red Meat | 0.592 | < 0.0005 |
| Eggs | -0.039 | 0.11 |
| Chicken | 0.341 | < 0.01 |
| Chicken Parts | 0.211 | < 0.01 |
| Tuna | 0.299 | < 0.01 |
| Other Fish | 0.368 | < 0.01 |
| Other Animal Parts | 0.211 | < 0.01 |
| Dairy Products Group | **0.309** | **< 0.01** |
| Cheese | 0.064 | 0.43 |
| Yogurt | 0.311 | < 0.05 |
| Milk | 0.279 | < 0.01 |
| Curd | 0.077 | 0.98 |
| Dough | 0.127 | 0.77 |
| Vegetables Group | **0.311** | **< 0.01** |
| Salad Vegetable | 0.288 | < 0.01 |
| Glandular Vegetables | 0.023 | 0.23 |
| Leafy Greens | 0.298 | < 0.01 |
| Potato | -0.433 | < 0.01 |
| Others | 0.297 | < 0.01 |
| Fruits Group | **0.568** | **< 0.01** |
| Apples | 0.371 | < 0.01 |
| Oranges | 0.381 | < 0.01 |
| Tangerine | 0.366 | < 0.01 |
| persimmons | 0.469 | < 0.01 |
| Pomegranates | 0.401 | < 0.01 |
| Banana | 0.599 | < 0.01 |
| Kiwi | 0.502 | < 0.01 |
| Apricots | 0.388 | < 0.01 |
| Cherries | 0.439 | < 0.01 |
| Grapes | 0.426 | < 0.01 |
| Peach | 0.501 | < 0.01 |
| Melons, Watermelon, Cantaloupe | 0.207 | < 0.01 |

NS, not significant

**Supplemental Table 3**

**Consumption frequency of food groups/ items related to whether subjects could afford to eat balanced meals**

| **Could not afford balanced meals** | **Total**  **n (%)** | **Consumed ≥ 5 days per week** | | | | **Consumed at least weekly** |
| --- | --- | --- | --- | --- | --- | --- |
|  |  | **Fruits**  **n (%)** | **Vegetables**  **n (%)** | **Dairy Products**  **n (%)** | **Rice**  **n (%)** | **Meat**  **n (%)** |
| Often | 67(13.4) | 17 (25.3) | 31 (46.2) | 38 (56.7) | 22 (32.8) | 9 (13.4) |
| Sometimes | 272 (54.4) | 174 (63.9) | 184 (67.6) | 209 (76.8) | 204 (75) | 163 (59.9) |
| Never | 161(32.2) | 136 (84.4) | 130 (80.7) | 141 (87.5) | 134 (83.2) | 136 (84.4) |
| P for trend |  | <0.005 | <0.01 | <0.01 | <0.005 | <0.001 |

Values were adjusted for age and sex
